# Socio-emotional skills profiles and their relations with career exploration and perceived parental support among 8th grade students

**DOI:** 10.3389/fpsyg.2023.1214395

**Published:** 2023-08-09

**Authors:** Vítor Gamboa, Suzi Rodrigues, Filipa Bértolo, Beatriz Marcelo, Olímpio Paixão

**Affiliations:** ^1^Faculty of Human and Social Sciences, University of Algarve, Faro, Portugal; ^2^Research Center for Psychological Science, University of Lisbon, Lisbon, Portugal

**Keywords:** career exploration, parental support, socio-emotional skills, latent profile analysis, person-centered approach

## Abstract

Socio-emotional skills can play a crucial role in students career development. This study used a person-centered approach to explore socio-emotional skills (curiosity, optimism, empathy, sociability, and responsibility) profiles among 8° grade students (*N* = 310). We also explored the relations of these profiles with career exploration (self and environmental), perceived parental support (emotional support, instrumental assistance, career-related modeling, and verbal encouragement) and school achievement. Using Latent Profile Analysis (LPA), four distinct profiles emerged that differed in terms of level and shape, namely: Other and Task oriented profile, Socio-emotional Adaptive profile, Socio-emotional non-Adaptive profile, Self- Oriented profile. Our results show that the “Socio-emotional Adaptive” profile can be clearly differentiated from the “Socio-emotional non-Adaptive” profile given the higher values it presents regarding all the variables in study. However, the differences between the “Other and Task Oriented” profile and “Self-Oriented” profile (intermediate profiles) were analyzed and discussed from qualitative point-of-view and adopting an exploratory approach. Overall, the findings of this study indicate that socio-emotional profiles have the potential to account for variations in career behaviors and academic performance. These results provide valuable insights for the development and implementation of career-oriented interventions targeted at 8th grade students and their immediate relational environments.

## 1. Introduction

Despite the observed differences in terminology and assessment (Schoon, 2021), nowadays it is consensual that socio-emotional skills are critical for positive development in education and career domains (Kidd, 2004; OECD, 2015). Long considered as barriers to be avoided, the advent of constructivist (e.g., Career Construction Theory; Savickas, 2005) and contextualist (e.g., Young et al., 1996) career approaches recognized the relevant role of emotions in career development, i.e., emotions and socio-emotional skills can in fact favor a successful resolution of vocational tasks and the progress in career decision-making (Hartung, 2011; Howard and Ferrari, 2021). However, despite the theoretical expectation that socio-emotional skills clearly affect vocational behavior (e.g., career exploration) and career development, to our knowledge, empirical research is scarce and has not yet produced consistent and robust results. Additionally, the study of emotions and socio-emotional skills has been mainly conducted through variable centered approaches, which only considers linear associations between the variables in study.

Similarly, career literature reports a vast number of studies that also tend to choose a variable-centered approach (Hofmans et al., 2020). Nevertheless, as argued by Morin et al. (2011), this approach does not allow to consider that results might differ between participants and between contexts. In fact, the work of Hofmans et al. (2020) highlights that the reality cannot always be simplified through the isolated analysis of relationships between variables, which assumes population-homogeneity. Thus, they propose the person-centered approach to fill this gap and to enrich the knowledge that can be extracted from variable-centered approaches (Meyer and Morin, 2016). Additionally, this approach has the potential to help researchers to: (1) better understand the impact of distinct combinations of multiple variables, which cannot be conveniently represented using other techniques that rely on the interaction of variables among a single population distribution (Zyphur, 2009); and (2) analyze individual differences, which can lead to distinct career interventions according to the specific needs of distinct groups (Wang and Hanges, 2011; Hofmans et al., 2020).

In the field of career development, we must also consider that adolescents are not a homogenous population regarding career behavior and coping strategies (e.g., Germeijs and Verschueren, 2006; Gamboa et al., 2014; Paixão and Gamboa, 2017). According to previous research, a valid way for the field of career development research to conceptualize the heterogeneity of students (e.g., in terms of career exploration, perceived parental support) is the identification of clusters of students who display similar patterns of career behavior (Brown and Ryan-Krane, 2000; Hofmans et al., 2020). In this sense, vocational psychology (e.g., Vondracek et al., 1986; Lent et al., 2002) and human motivation theories (e.g., Ryan and Deci, 2000) have come to acknowledge the importance of a differentiated approach to the investigation and management of career issues. When it comes to adolescents, profiles based on socio-emotional skills have rarely been investigated and little is known about how these profiles explain differences in career exploration behaviors, perceived parental support and school achievement. From our point of view, investigating differences in students’ career behavior (e.g., career exploration), considering distinct socio-emotional profiles, can be particularly insightful, as these constructs are central to promoting adaptive career trajectories (e.g., Paixão and Gamboa, 2017). Furthermore, knowledge on students’ socio-emotional profiles is also essential for career interventions delivered in school contexts.

### 1.1. Socio-emotional skills

Socio-emotional skills can be defined as the ability to regulate thoughts, emotions, and behaviors, being considered a malleable construct that can be developed through formal and informal learning experiences, in school and family contexts (Kankaraš and Suarez-Alvarez, 2019). According to the literature, the development of socio-emotional skills is related with individuals’ development (Malti and Noam, 2016), well-being (Chernyshenko et al., 2018; Jagers et al., 2019), academic achievement (Weissberg et al., 2015), and employment (Howard and Ferrari, 2021). In other words, we can argue that socio-emotional skills are especially important for success at school and in life. Therefore, socio-emotional skills can and should be taught, modeled, practiced, and applied to different situations in order to be used by individuals as part of more adaptive behaviors (Weissberg et al., 2015). Adolescence, as well as childhood, is a life-stage where we can observe a significant development of these types of self-regulatory skills (e.g., responsibility, optimism, curiosity), which can play a crucial role in how the individual deal with vocational tasks (e.g., the transition to secondary education) (Chernyshenko et al., 2018).

Across literature we can also notice that socio-emotional skills are commonly related to the Big Five personality traits framework. The use of this multidimensional framework in distinct psychology research subareas, allows the distinction of socio-emotional variables between skills, personality traits, thoughts, behaviors, and other related constructs (Danner et al., 2021). Hence, in our study we opted to use five socio-emotional skills (OECD, 2021a,b) aligned with each one of the Big Five framework dimensions (John et al., 2008), namely: Responsibility (Conscientiousness), Optimism (Neuroticism), Sociability (Extraversion), Empathy (Agreeableness), and Curiosity (Openness).

In order to advance with the differentiation of socio-emotional skills from related constructs, Schoon (2021) proposes an integrative taxonomy of domains and manifestations of socioemotional competences (DOMASEC), in which she states that socio-emotional skills can be addressed to each one of the Big Five dimensions and categorized as being self-oriented, other-oriented, and task-oriented. Thus, according with this taxonomy we are considering responsibility and optimism as self-orientation skills, sociability and empathy as other-oriented skills and curiosity as task-oriented skills. Schoon (2021) also considers that the nature of each skill allows the possibility to make a parallelism with the intra - and interpersonal dichotomy used by other authors (e.g., Bar-On, 1997; Di Fabio and Kenny, 2015). Intrapersonal skills would be associated with the individual ability to express its feelings, as well the awareness about its own emotions, strengths, and weaknesses (self-oriented). Alternatively, interpersonal skills are usually linked to social awareness and the ability to establish and maintain cooperative, constructive, and satisfactory interpersonal relationships (other-oriented).

### 1.2. Socio-emotional skills, career exploration and parental support

According to Howard and Ferrari (2021), the growing interest in the relationship between socio-emotional skills and career development stems from the idea that managing emotions facilitates exploration and progress in career decision-making. Thus, in complex vocational tasks, such as choosing a secondary school course, socio-emotional skills become more important as students must deal with stress and ambiguity associated with career exploration and career decision-making. Also, in these type of academic transitions students may also benefit from the security and structure that is provided by parental support (Kenny and Medvide, 2013; Katz et al., 2018), since it can be crucial to enhance career exploration behaviors (Turan et al., 2014; Guan et al., 2015) and career adaptive behaviors (e.g., Whiston and Keller, 2004; Hartung et al., 2005). In other words, students with higher levels of socio-emotional skills are less likely to drop out when facing difficulties during career exploration activities (Di Fabio et al., 2012; Di Fabio and Kenny, 2015). Furthermore, according to contextual action theory of career development (Young et al., 1996), emotions play a crucial role in constructing one’s career through everyday actions. For example, the role of emotions in career construction was studied by Young et al. (1997), who resort on the dialogs of 14 parent–child dyads, concluding that emotions have a regulating function in the collaborative development of career projects.

Therefore, we can expect that students with higher levels of socio-emotional skills might tend to seek more parental support on career-related issues.

Overall, despite the small number, it is possible to observe a gradual growth of studies that seek to study the role of socio-emotional variables on career behaviors (Kidd, 2004; Hartung, 2010, 2011). Most of these studies use emotional intelligence as an intrapersonal socioemotional competence, which seems to be positively associated to adaptive career behaviors (Pirsoul et al., 2022), career choice (Di Fabio and Kenny, 2011) and career decision-making self-efficacy (Di Fabio and Palazzeschi, 2008), and negatively associated to career decision-making difficulties (Di Fabio and Palazzeschi, 2009b), career indecision and indecisiveness (Di Fabio et al., 2013). Using the Career Construction Theory (CCT, Savickas, 2013), the studies of Parmentier et al. (2019) and Parmentier et al. (2022) deepened the extension of indirect relationships between these variables and found positive associations between emotional intelligence and career adaptability and, in turn, with career decision-making self-efficacy. The work of Mittal (2021) and Nieto-Flores et al. (2019), also through career adaptability, found results that suggest positive associations between emotional intelligence and job-search behavior and job-search self-efficacy. In general, the role of emotions and their use as a self-regulation strategy seem to be positively associated to career construction (Young et al., 1997), and negatively to university indecisiveness (Farnia et al., 2018) and amount of occupational information (Santos et al., 2018). Finally, by using the Big Five model of personality as main framework, Di Fabio and Palazzeschi (2009b) found a negative correlation between career difficulties and extraversion and positive with neuroticism, and Li et al. (2015) present openness to experience, agreeableness, and conscientiousness as predictors of higher levels of career exploration. In summary, we can infer that socio emotional skills are positively associated with career exploration and perceived parental support.

### 1.3. Socio-emotional skills and school achievement

Generally, socio-emotional skills have been considered crucial for school achievement (Weissberg et al., 2015). Empirical research revealed that whether being considered as resources, competences, skills, or behaviors, socio-emotional variables seem to be positively related to school variables (directly and indirectly), namely: school readiness (Duncan et al., 2007), school achievement (Sudkamp et al., 2017), and academic well-being (Duncan et al., 2007; Durlak et al., 2011; Wilkins et al., 2015; Sudkamp et al., 2017; Roberts et al., 2018). Moreover, socio emotional skills can be considered protective factors against school drop-out and can promote academic engagement (Salmela-Aro and Upadyadya, 2020). The work of Durlak et al. (2011) provided empirical evidence on the impact of socio-emotional skills on school achievement. In a meta-analysis that included 213 school-based Socio Emotional Learning.

(SEL) programs, SEL participants demonstrated a significant improve on social and emotional skills and academic performance, when compared to controls. Overall, Durlak et al. (2011) acknowledges socio-emotional learning programs as a promising approach that enhances children’s success in school and in life. More recently, Chernyshenko et al. (2018) highlight that socio-emotional skills can have direct and indirect effects on school outcomes. For example, being curious and open-minded and having an active approach toward learning is an important pre-requisite for developing and improving innate cognitive capacities. Additionally, empathy can also be helpful to children’s adaptation to the school environment, to gain higher status among their peers and, consequently, to achieve better academic results.

### 1.4. The present study

Using person-centered approach, the present study aims to, firstly, differentiate profiles based on socio-emotional skills (OECD, 2021a,b). Secondly, considering the outcomes that we have found in literature we intend to analyze the differences between the emerged profiles regarding career exploration, perceived parent support and school achievement, in order to conceptualize and discuss them.

To our knowledge, research that intent to conceptualize profiles based on socio-emotional variables are very scarce and vary on the theoretical framework on its basis. Whitin these studies we can observe that commonly two profiles are extracted, being typically labeled based on their high or low socio-emotional levels. Also, a third profile regularly emerges as an intermediate profile and is analyzed with an exploratory approach given that their levels are not as theoretically normative as the rest of the profiles (e.g., Sudkamp et al., 2017; Castro-Kemp et al., 2019; Pulido-Martos et al., 2022). For example, Castro-Kemp et al. (2019) research organized the profiles based on four socio-emotional variables: gratitude, optimism, zest, and persistence. Here they found that the profile with lowest levels of optimism presented lower levels of socioemotional health and greater emotional and behavioral school-related difficulties. The work of Pulido-Martos et al. (2022) self-esteem and emotional intelligence are considered as socioemotional resources. They found a profile with low levels socio-emotional resources that also presented the lowest levels of perceived social support (from parents, peers, and teachers), self-emotional and others appraisal and emotional regulation. Contrarily, a profile with high socioemotional resources emerged with high levels of the mentioned variables. Sudkamp et al. (2017) conceptualized their profiles based on cognitive (IQ) and socio-emotional competences (academic self-concept, academic motivation, and achievement-related anxiety) and realized that the profile with higher levels of socio-emotional competences is the one that also shows higher IQ levels and the best school achievement when compared to the rest of the groups. In contrast, the group with lower socio-emotional competences present lower IQ and low school achievement.

In this study we use Latent Profile Analysis (LPA) to address our first goal, which is a method commonly used among person-centered approaches in career research. It consists in a categorical latent variable modelling approach that aims to identify subpopulations (latent profiles) within a population, based on a similarity pattern that they share among a certain set of variables (LPA indicators). According to Spurk et al. (2020), in the past decade we can observe an increment in the use of this method in the study of career-related variables. However, despite the potential to address specific research questions and to expand theoretical knowledge regarding career predictors and outcomes, as well as the individual’s heterogeneity among career subjects, variable-centered approach still predominates and the application of LPA is still very scarce (Hirschi and Valero, 2015; Gillet et al., 2018; Hofmans et al., 2020; Spurk et al., 2020).

We could expect the emergence of two distinct socio-emotional profiles, that according to the evidence should be clearly distinguishable in quantity, i.e., a profile with higher levels of socio-emotional skills associated to higher levels on career exploration behaviors, higher levels on perceived parental support, higher grades, and lower school failure percentage than the other profile. In other words, these two socio-emotional profiles will display significant differences in terms of career exploration, parental support, and school achievement. However, the empirical research we analyzed on socio-emotional skills and vocational behavior show solutions with three or more profiles (e.g., Parmentier et al., 2022; Pirsoul et al., 2022), identifying groups with intermediate levels of socio-emotional skills, which might present less predictable levels for career-related and school achievement variables. For these intermediate profiles we adopted an exploratory approach to their conceptualization and respective discussion.

## 2. Methods

### 2.1. Participants and procedure

The sample comprises 310 students, being 163 males (52.6%) and 147 females (47.4%) with ages between 13 and 15 years old (*M* = 13.38, SD = 0.62), from six public schools in the southern Portugal. In addition to this demographic information, we also collected academic achievement data, namely the grades in Portuguese and Math subjects (1–5 points) and whether the students failed any course of study (school failure rate). For the Portuguese subject grades varied between 2 and 5, being the mean value 3.25 (SD = 0.69) and for Math grades the values ranged from 1 to 5 and the mean value was 3.17 (SD = 0.92). Regarding school failure rate, 24.5% said that they have failed one or more times and 75.5% replied that they had not failed until the date of data collection.

### 2.2. Procedure

The study was presented to schools in an initial phase and the appropriate informed consent procedures and permissions were gathered from parents and school board. Data collection was made by trained coresearchers in classroom context, with the assistance of the school psychologist. Participants were informed about the general subject of the study and that their participation was voluntary and confidential. On average, each assessment required 25 min.

### 2.3. Measures

We assessed socio-emotional skills with the Portuguese version of Socio-Emotional Skills Survey (SSES; OECD, 2021a,b), provided by Calouste Gulbenkian Foundation, which aims to better understand students’ contextual factors (e.g., school, home, community) and the characteristics that directly or indirectly influence the development of social and emotional skills. The SSES conceptual framework is based on the OECD framework (Chernyshenko et al., 2018; Kankaraš and Suarez-Alvarez, 2019) and was developed in reference to the ‘Big Five Model’ (John et al., 2008) that distinguishes 15 skills in five dimensions: (1) Task Performance (self-control, responsibility, persistence); (2) Emotional Regulation (stress resistance, optimism, emotional control); (3) Collaboration (empathy, trust, cooperation); (4) Open-Mindedness (tolerance, curiosity, creativity); (5) Engaging with Others (sociability, assertiveness, energy). In this study, we used responsibility, optimism, empathy, curiosity, and sociability as the skills that represent each dimension, respectively. The validity and reliability of the scale have been demonstrated in other studies, reporting Cronbach’s alpha that ranged from 0.80 and 0.81 (e.g., Kankaraš and Suarez-Alvarez, 2019; Salmela-Aro and Upadyadya, 2020). In this study, internal consistency value for the scale was 0.94.

The perceived parental support was assessed with the *Career-Related Parent Support Scale* (CRPSS, Turner et al., 2003; adapt. Gamboa et al., 2021). It aims to assess students’ perceptions of parental support toward career and educational development along the four sources of self- efficacy expectations proposed by Bandura (1997). The scale comprises 27 items distributed among four subscales: (1) Instrumental Support (6 items, e.g., “My parents help me to choose out-of-school activities that may be useful in my future professional career”); (2) Career Modelling (7 items, e.g., “My parents have already shown me where they work”); (3) Verbal Persuasion (5 items, e.g., “My parents praised me for doing my schoolwork well”); and (4) Emotional Support (6 items, e.g., “My parents say they are proud of me when I am successful in school”). Items were rated using a 5-point Likert-type scale (1 – strongly disagree to 5 – strongly agree), and higher scores represent greater perceived parental support. The validity and reliability of the scale was demonstrated both in the original version (Turner et al., 2003), and in the Portuguese version (Gamboa et al., 2021). In our study, Cronbach Alpha values for the total scale was 0.93, while for the subscales the values varied between 0.79 (Verbal Encouragement) and 0.88 (Emotional Support).

Career exploration was assessed using the Portuguese version of the Career Exploration Survey (CES; Stumpf et al., 1983; adapt. Taveira, 1997). The CES is a multidimensional self- administered survey with 53 items (Likert-type response format), designed to assess beliefs, processes, and reactions to career exploration. We only used the items that compose two processes of career exploration: Self-Exploration (5 items, e.g., “In the last 3 months I reflected on how my past integrates with my future career”) and Environmental Exploration (4 items, e.g., “In the last 3 months I went to various career orientation programs”). The validity, reliability, and multidimensionality of the CES have been widely demonstrated in its’ different versions. Cronbach’s alpha for the Portuguese version ranged from 0.63 to 0.83. Research that used this version found internal consistency values for the two exploration processes used in this study between 0.74 and 0.79 (Paixão and Gamboa, 2017, 2022; Gamboa et al., 2021), which is aligned with the values we found in our study (Environmental Exploration = 0.81; Self- Exploration = 0.77).

### 2.4. Analysis

In the first step, we computed the means, standard deviations, correlations, and internal consistency for the variables in study. Secondly, we performed Latent Profile Analysis (LPA) in Jamovi (2.3.21) software to identify profiles based on the chosen socio-emotional variables. Normality of each profile was tested using the criteria mentioned by Marôco (2010). Bootstrapped likelihood ratio test (BLRT), Akaike Information Criterion (AIC), Bayesian Information Criterion (BIC), Sample size-adjusted BIC (SABIC), and Entropy were used to determine the optimal number of profiles. With exception made for Entropy, lower values of these criteria indicate better model fit and parsimony (Nylund et al., 2007). Entropy values range from 0 to 1 and, contrarily to the other used criteria, the higher the value the better differentiations between profiles (Celeux and Soromenho, 1996) and values between 0.60 and 0.80 are considered as appropriate (Muthén, 2004; Jung and Wickrama, 2008). Furthermore, significant *p*- values for BLRT means that the current k-class model fits better than the model with k + 1 classes (Nylund et al., 2007). Finally, profile membership was used in a multivariate analysis of variance (MANOVA), and through post-hoc tests we examined differences between the socio-emotional profiles (independent variables) regarding career exploration behaviors, perceived parental support and Portuguese and Math’s grades (dependent variables).

## 3. Results

Table 1 shows the descriptive and correlation results between variables in study. Overall, we can observe positive and significant correlations between almost all variables in study, with exception for the one between responsibility and environmental exploration. Concerning academic achievement variables, Portuguese grades presented positive association with all variables except with sociability and environmental exploration. Math grades only seem to be positively correlated to responsibility (*r* = 0.35, *p* < 0.01), curiosity (*r* = 0.15, *p* < 0.01), verbal encouragement (*r* = 0.17, *p* < 0.01), and Portuguese grades (*r* = 0.59, *p* < 0.01). Cronbach alpha values are within those that literature presents as adequate (Marôco, 2010).

**Table 1:** Means, standard deviation, internal consistency, and bivariate correlations between all variables in study (*N* = 310).

	***M***	**DP**	**1**	**2**	**3**	**4**	**5**	**6**	**7**	**8**	**9**	**10**	**11**	**12**	**13**
1. Responsibility	3.40	0.61	(0.75)	0.35^**^	0.20^**^	0.31^**^	0.42^**^	0.10	0.25^**^	0.27^**^	0.29^**^	0.29^**^	0.32^**^	0.34^**^	0.35^**^
2. Optimism	3.68	0.78		(0.86)	0.51^**^	0.27^**^	0.35^**^	0.15^**^	0.22^**^	0.40^**^	0.36^**^	0.27^**^	0.41^**^	0.13^**^	0.09
3. Sociability	3.72	0.71			(0.81)	0.43^**^	0.40^**^	0.29^**^	0.25^**^	0.42^**^	0.34^**^	0.31^**^	0.35^**^	0.05	−0.02
4. Empathy	3.69	0.55				(0.72)	0.55^**^	0.27^**^	0.39^**^	0.45^**^	0.32^**^	0.41^**^	0.41^**^	0.24^**^	0.07
5. Curiosity	3.87	0.65					(0.83)	0.20^**^	0.39^**^	0.41^**^	0.38^**^	0.33^**^	0.44^**^	0.30^**^	0.15^**^
6. Environmental exploration	2.81	1.06						(0.81)	0.63^**^	0.42^**^	0.42^**^	0.29^**^	0.25^**^	0.06	0.01
7. Self-exploration	3.16	0.95							(0.77)	0.52^**^	0.46^**^	0.31^**^	0.39^**^	0.13^**^	0.10
8. Emotional support	3.74	0.95								(0.88)	0.79^**^	0.55^**^	0.75^**^	0.19^**^	0.04
9. Instrumental assistance	3.63	0.86									(0.80)	0.55^**^	0.72^**^	0.25^**^	0.09
10. Career-related modeling	4.16	0.75										(0.82)	0.56^**^	0.22^**^	0.03
11. Verbal encouragement	4.31	0.69											(0.79)	0.34^**^	0.17^**^
12. Portuguese grade	3.25	0.69													0.59^**^
13. Math grade	3.17	0.92													

*p < 0.05; **p < 0.01. Internal consistency for each variable is presented between parentheses.

We performed LPA using responsibility, optimism, sociability, empathy, and curiosity as latent profile indicators. To ensure that all measures contributed equally to the analysis, we standardized the original mean values to generate a set of z-scores (*M* = 0, SD = 1). To determine the optimal number of latent profiles, we followed a stepwise approach starting with a solution with two profiles and successively looking the fit indices regarding a solution with one more profile (Nylund et al., 2007). Also, we tried to ensure that none of the profiles presented a total of subjects that could be considered too low (less than 3%), so that we could be able to find statistically significant differences between profiles for each variable, and that each one could be theoretically relevant and meaningful (Marôco, 2010; Spurk et al., 2020). Considering these criteria, we opted for the 4-profile solution which presented adequate values for AIC (6782), BIC (6901), SABIC (6812) and significant BLRT (62.27; *p* < 0.05) and an Entropy value (0.74).

**Figure 1:**
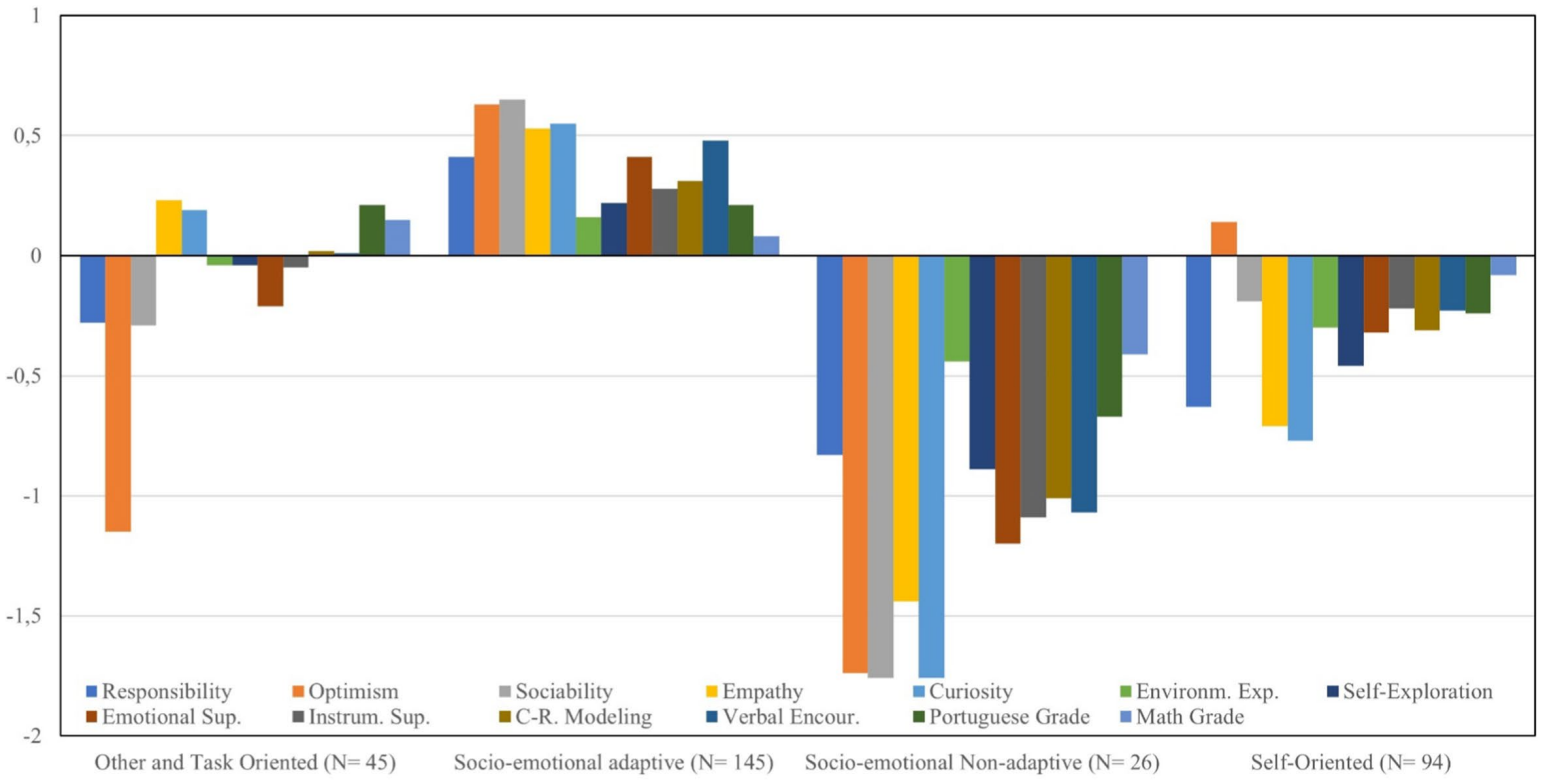
Profile results based on *z*-scores of the socio-emotional skills and criterion variables for the four profile solution.

Figure 1 shows the graphical distribution of each profile according to the respective z-scores for each variable in study. The first profile (*N* = 45, 14.5%) was labeled as “Other and Task- Oriented.” Concerning the socio-emotional skills, this group is characterized by z-score values below the mean value for responsibility (−0.28), optimism (−1.15) and sociability (−0.29) and above the mean value for empathy (0.23) and curiosity (0.19). It also presents values below the mean value for environmental exploration (−0.04) self-exploration (−0.04), emotional support (0.21) and instrumental support (−0.05). Finally, shows positive z-scores for career-related modeling (0.02), verbal encouragement (0.01), Portuguese grades (0.21) and the highest value for Math grades (0.15). Also, this profile presents a school failure of 22.2% (the second lowest among all profiles).

The second profile (*N* = 145, 46.8%), which includes the largest number of participants, was labeled as “Socio-emotional Adaptive,” as it shows the highest z-score levels for all socio- emotional variables. Concerning the remaining variables it also presents the highest values among all profiles, except for Portuguese grade (0.21), which is equal to the first groups value, and Math grades (0.08). Additionally, this was the profile with the lowest school failure rate (20.7%).

The third profile (N = 26, 8.4%) was labeled as “Socio-emotional non-Adaptive” and has the smallest number of participants. This group is characterized by the lowest z-scores among all socio-emotional variables when compared with the other extracted profiles. Moreover, regarding dependent variables, this profile shows the lowest z-scores and the highest school failure rate (46.2%).

Lastly, the fourth profile (*N* = 94, 30.3%) was labeled as “Self-Oriented” as it reveals z- scores values below the mean value for all socio-emotional variables except for optimism (0.14). This group also presents z-scores above the mean value for the remaining variables in study and a school failure rate of 25.5%.

In the next step, we performed chi-squared test to examine whether there was any relationship between student’s gender and their profile. A significant relationship was found (*χ*^2^ (3) = 14.1, *p* < 0.05), so we controlled this variable in the subsequent analyses. By performing univariate analysis of covariance, we can observe that the profiles differ significantly for all variables in study. Finally, we conducted *post hoc* Tukey’s honestly significant difference tests to examine with better detail how the groups differed regarding career exploration, perceived parental support and Portuguese and Math’s grades.

Table 2 shows global means, standard deviations, and z-scores for the four-profile solution, as well the number of subjects for each group and their respective school failure rate. It also shows the results of the analyses of variance that were performed to determine the relative contribution of the different socio-emotional variables to the differentiation of the profiles, as well as the corresponding effect size (*η*^2^). The variables that contributed the most to discrimination among the groups were optimism, *F* (3, 310) = 168.1, *p* < 0.01, *η*^2^ = 0.62, and curiosity, *F* (3, 310) = 106.2, *p* < 0.01, *η*^2^ = 0.51.

**Table 2:** Means, standard deviations, and *z*-scores for the Latent profiles and criterion variables in the four profiles (*N* = 310).

	**Group 1 (*N* = 45)**			**Group 2 (*N* = 145)**			**Group 3 (*N* = 26)**			**Group 4 (*N* = 94)**			***F***	***p***	***η*** ^**2**^
	***M***	**(DP)**	***z***	***M***	**(DP)**	***z***	***M***	**DP**	***z***	***M***	**DP**	***z***			
Responsibility	3.29^a^	(0.54)	−0.28	3.73^b^	(0.56)	0.41	2.95^c^	(0.58)	−0.83	3.07^a,c,d^	(0.74)	−0.63	37.4	0.00	0.27
Optimism	2.77^a^	(0.45)	−1.15	4.15^b^	(0.45)	0.63	2.31^c^	(0.66)	−1.74	3.77^d^	(0.63)	0.14	168.1	0.00	0.62
Sociability	3.47^a^	(0.57)	−0.29	4.13^b^	(0.51)	0.65	2.44^c^	(0.63)	−1.76	3.55^a,d^	(0.71)	−0.19	89.0	0.00	0.47
Empathy	3.84^a^	(0.36)	0.23	4.01^a,b^	(0.44)	0.53	2.92^c^	(0.50)	−1.44	3.32^d^	(0.61)	−0.71	89.2	0.00	0.46
Curiosity	4.04^a^	(0.39)	0.19	4.26^b^	(0.40)	0.55	2.84^c^	(0.69)	−1.76	3.45^d^	(0.72)	−0.77	106.2	0.00	0.51
Environm. Exp.	2.83^a^	(1.05)	−0.04	3.03^a,b^	(1.07)	0.16	2.41^a,c^	(1.11)	−0.44	3.56^a,c,d^	(0.97)	−0.30	6.3	0.00	0.06
Self-Exp.	3.23^a^	(0.93)	−0.04	3.48^a,b^	(0.84)	0.22	2.45^c^	(0.99)	−0.89	2.84^a,c,d^	(0.96)	−0.46	16.5	0.00	0.14
Emotional Sup.	3.58^a^	(0.99)	−0.21	4.14^b^	(0.68)	0.41	2.67^c^	(1.14)	−1.20	3.48^a,d^	(0.92)	−0.32	28.7	0.00	0.22
Instrum. Sup.	3.61^a^	(0.77)	−0.05	3.90^a,b^	(0.74)	0.28	2.73^c^	(1.06)	−1.09	3.47^a,d^	(0.91)	−0.22	18.2	0.00	0.15
C-R. Modeling	4.20^a^	(0.73)	0.02	4.41^a,b^	(0.63)	0.31	3.42^c^	(0.95)	−1.01	3.94^a,d^	(0.93)	−0.31	19.9	0.00	0.16
Verbal Encour.	4.26^a^	(0.75)	0.01	4.61^b^	(0.42)	0.48	3.47^c^	(0.92)	−1.07	4.09^a,d^	(0.88)	−0.23	33.1	0.00	0.25
Portuguese grade	3.40^a^	(0.65)	0.21	3.39^a,b^	(0.72)	0.21	2.79^c^	(0.66)	−0.67	3.09^c,d^	(0.57)	−0.24	8.72	0.00	0.08
Math grade	3.31^a^	(0.87)	0.15	3.24^a,b^	(0.94)	0.08	2.79^a,b,c^	(0.98)	−0.41	3.10^a,b,c,d^	(0.89)	−0.08	2.20	0.08	0.02
													χ^2^	*p*	
Gender													14.1	0.00	
Male/Female (N)	14/31			75/70			13/13			61/33					
Male/Female (%)	31/69			52/48			50/50			64/36					
School failure (%)	22.2			20.7			46.2			25.5					

The results reveal that the “Socio-emotional adaptive” group and the “Socio-emotional nonadaptive” profile differed significantly at all variables except for Math’s grades. The comparison between “Socio-emotional adaptive” profile and the “Other and task-oriented” group shows significant differences for emotional support, verbal encouragement and for all socio-emotional skills, except for empathy. When comparing “Socio-emotional adaptive” group the “Self-Oriented” group, significant differences can be seen for all variables in study, with exception for Math’s grades. “Socio-emotional non-adaptive” profile is significantly different at all variables except for environmental exploration and Math’s grade from “Other and Task- oriented group.” When compared with “Self-Oriented” profile, the “Socio-emotional nonadaptive” group shows differences for all parent support variables and for all socio- emotional skills, except responsibility. Finally, “Other and task-oriented” profile and “Self- Oriented” group reveal significant differences for optimism, empathy, curiosity, and Portuguese grades.

## 4. Discussion

To our knowledge research that conceptualized socio-emotional profiles are very scarce, and the number of studies that tried to assess the differences of such profiles among career-related variables are even fewer. The present study had a twofold purpose. Firstly, we used LPA to identify profiles based on socio-emotional skills measured with the SSES (OECD, 2021a,b), which was developed in reference to the ‘Big Five Model’ (John et al., 2008). Second, we sought to verify how the profiles would differentiate among exploration behaviors (environmental exploration and self-exploration), perceived career-related parent support, and academic achievement (school failure rate, Portuguese, and Math grades).

A four-profile solution was found to be the one that gathered the best consensus concerning the evaluated psychometric properties and the followed theoretical assumptions.

As expected, LPA revealed heterogeneity within the sample. A group of students emerged with the highest levels of all socio-emotional skills, career exploration and perceived parental support among all profiles, being labeled as “Socio-emotional Adaptive.” In other hand, a profile of students with the lowest levels across all the variables also emerged and was labeled as “Socio-emotional non-Adaptive.” In fact, we can find evidence in literature for the positive association between socio-emotional variables and career adaptive behaviors and processes in literature, such as career adaptability (e.g., Parmentier et al., 2019), job-search self-efficacy (e.g., Nieto-Flores et al., 2019), career choice (e.g., Pirsoul et al., 2022), career decision-making self-efficacy (e.g., Di Fabio and Palazzeschi, 2009b; Di Fabio and Kenny, 2011) and negative association to career indecision (e.g., Di Fabio et al., 2013). Additionally, the school achievement values for these two profiles corroborate the suggestions of studies that associate high levels of socio-emotional skills with higher school achievement (Sudkamp et al., 2017), and lower levels to academic difficulties (Castro-Kemp et al., 2019). Thus, “Socio-emotional Adaptive” is the group with the least percentage of school failure and has Portuguese and Math grades above the mean value. Contrarily, the “Socio-emotional non-Adaptive” group shows up as the group with the poorer school achievement and the highest percentage of school failure among all the emerged profiles.

The remaining two profiles found do not present a combination of socio-emotional skills and career-related levels as theoretically normative as those described above, and therefore, we adopted an exploratory approach to conceptualize and to compare them. First, we obtained a group of students with levels above the mean value for empathy and curiosity. Inspired by the DOMASEC taxonomy (Schoon, 2021), we decided to label it as the “Other and Task Oriented” profile. The other profile emerges as having positive z-scores for optimism. By comparing these two profiles, we can observe that the “Other and Task Oriented” group presents higher levels of career exploration and perceived parental support than the “Self-Oriented” group. They also differ regarding school achievement, since that “Self-Oriented” profile shows poorer grades in Portuguese and Math subjects and a percentage of school failure higher than the “Other and Task Oriented” profile. From a qualitative perspective the “Other and Task Oriented” profile corroborates the results obtained by Li et al. (2015), who found positive associations between conscientiousness, agreeableness, and openness to exploration behaviors. The study of Chernyshenko et al. (2018) also suggested a positive association between curiosity and the improvement of cognitive capacities, which can lead to better academic outcomes. In our study we used responsibility, empathy, and curiosity to represent, respectively, these three dimensions of the Big Five framework (John et al., 2008). The results of the “Self-Oriented” profile can also be in accordance with literature if we consider optimism as the socio-emotional skill aligned with neuroticism, which is considered an intrapersonal and Self-Oriented skill (e.g., Bar-On, 1997; Di Fabio and Kenny, 2015; Schoon, 2021). Also, the neuroticism it can be also associated to an emotionally negative or instable individual, and nervousness (John et al., 2008). In fact, Di Fabio and Palazzeschi (2009a) found positive associations between neuroticism and career difficulties. Therefore, the statistically significant differences that the “Other and Task Oriented” profile present the “Self-Oriented” group regarding socio-emotional skills may be the reason why the first group obtains higher levels at the career-related and school achievement variables.

Finally, if we compare the “Other and Task Oriented” profile with the “Socio-emotional Adaptive” profile (the only profile with exploration levels above the mean value) we can observe that they differ at the responsibility, optimism and sociability levels. If we look at career exploration as a complex and goal-oriented process that implies some level of persistence and organization, we may consider that curiosity and empathy (as socio-emotional skills) may not be enough to lead exploratory behavior to more expressive levels. Thus, a broader set of socio- emotional skills may be needed to ensure higher levels of exploration. For example, responsibility, as a skill defined as the ability to honor commitments and be punctual and reliable (OECD, 2021a,b), could be an important self-regulatory competence to achieve greater exploration.

## 5. Theoretical and practical implications

The present study contributes to the development of person-centered approaches to the study of the influence of socio-emotional skills on vocational behaviors. Theoretically, our results reveal that it is possible to differentiate profiles based on the individual’s socio-emotional skills from a quantitative point of view (high and low levels of socio-emotional skills), but also from a qualitative perspective (intermediate levels of socio-emotional skills). This draws the attention to the fact that distinct combinations of skills might result in distinct vocational behaviors and, consequently, different career and academic outcomes. In sum, according to our results, we can conclude that adolescents are not a homogeneous group regarding socio-emotional skills and that socio-emotional profiles are able to explain significant differences in career behaviors and school achievement. However, the fact that we opted for a person-centered approach does not necessarily mean that this should replace variable-centered approaches, but rather complement each other, in order to provide greater robustness to the results and richness in their discussion (Wang and Hanges, 2011; Meyer and Morin, 2016).

The results can also offer insights to the conceptualization and preparation of vocational interventions, namely those which aim to involve the individual’s relational contexts in order to promote the development of socio-emotional skills at the service of career behaviors. In the educational context, for example, the results highlight the importance of developing a curriculum that combines socio-emotional skills and career education, helping students to develop skills in different domains (i.e., self-oriented, other oriented and task oriented) that, in turn, can enhance their vocational development.

From the career intervention viewpoint, “Socio-emotional Adaptive” students will benefit from less control from teachers and parents, and more autonomy in the exploration process. Also, great diversity of opportunities to explore occupational realities and to reflect about themselves should be considered. Globally, this will enhance these student’s perceptions of agency and authorship. In contrast, the “Socio-emotional Non-Adaptive” students, as the least favorable career profile, should benefit from highly structured career interventions. For example, career counselors should organize exploration activities in a step-by-step procedure with specific goals. Additionally, given the low values of socio-emotional skills presented by this group of students, socio-emotional learning interventions should be provided in order to support career adaptive development.

Finally, considering that a set of socio-emotional skills seems to be needed to reach more adaptive career behaviors (rather than isolated skills), the “Other and Task Oriented” and “Self- Oriented” profiles should also benefit from socio-emotional learning training. For example, these profiles should benefit from activities that promote curiosity, optimism and responsibility, such as, oriented exploratory activities with specific deadlines (organized with grids), followed by group sessions to debrief the gathered information.

In sum, the possibility to organize students by their socio-emotional skills leads us to reaffirm the importance of differential career interventions practices. These practices should be based in complete information regarding socio-emotional profiles of students and the quality of family and school contexts.

## 6. Limitations and future research

Although our study can represent a contribute to the positive aspects of person-centered approaches, there are some limitations that need to be underlined. One of them is directly related to LPA method, which seems to be sensitive to sample sizes and, consequently to the overextraction of profiles (Meyer and Morin, 2016; Spurk et al., 2020). This issue can be controlled using appropriate fit indices, as suggested by Muthén (2004) and Nylund et al. (2007). Being a multivariate exploratory method can also be a concern on the use of LPA. However, we suggest to not blindly follow statistical criteria on the extraction of the profiles and try to also ensure that there is not great disparity between the extracted profiles, and that they can be theoretically relevant and meaningful (Marôco, 2010).

Our results are limited to the role of socio-emotional skills on career behaviors, and, therefore, they should not be generalized to proximal constructs, such as emotional intelligence or socio-emotional learning. According to the suggestions of Howard and Ferrari (2021) and Schoon (2021), an integrative framework is needed to clearly differentiate the relationship that socio-emotional variables may have with career constructs, as either being skills, traits, or behaviors.

Future research should ensure the use of other indicators beyond self-reported measures, such as parents and teachers’ versions of socio-emotional skills, to clarify the extent to which these aspects are related to students’ socio-emotional and career profiles. Moreover, our study adopted a cross-sectional design, limiting our ability to make any inferences about the causal relations between the antecedents and outcomes of the socio-emotional profiles. Thus, in the future should focus on longitudinal studies in order to better investigate the developmental trajectories of each of socio-emotional profiles.

Finally, our study does not clearly consider career outcome variables, such as career indecision. Future research should include variables that can assume this role to take full advantage of the person-centered approach, which can lead to the development of assumptions that guide to confirmatory studies (Muthén, 2004).

## Data availability statement

The raw data supporting the conclusions of this article will be made available by the authors, without undue reservation.

## Ethics statement

The studies involving human participants were reviewed and approved by the data protection office of the University of Algarve and ethics committee of the University of Algarve. Written informed consent to participate in this study was provided by the participants’ legal guardian/next of kin.

## Author contributions

VG designed the study and wrote the first draft of the manuscript. All authors contributed to the study concept and design and discussed the data analyses, contributed to the article and approved the submitted version.

## Funding

The work was supported by the Fundação Calouste Gulbenkian, as part of their Gulbenkian Academies of Knowledge initiative, which financed the open access publication fees.

## Conflict of interest

The authors declare that the research was conducted in the absence of any commercial or financial relationships that could be construed as a potential conflict of interest.

## Publisher’s note

All claims expressed in this article are solely those of the authors and do not necessarily represent those of their affiliated organizations, or those of the publisher, the editors and the reviewers. Any product that may be evaluated in this article, or claim that may be made by its manufacturer, is not guaranteed or endorsed by the publisher.
